# Laparoscopic negative appendectomy during pregnancy is associated with adverse neonatal outcome

**DOI:** 10.1007/s00464-021-08315-2

**Published:** 2021-02-01

**Authors:** Misgav Rottenstreich, James Tankel, Naama Vilk Ayalon, Reut Rotem, Shlomo Yellinek, Fayez Khatib, Sorina Grisaru-Granovsky

**Affiliations:** 1 Department of Obstetrics & Gynecology, Shaare Zedek Medical Center, Affiliated with the Hebrew University School of Medicine, Jerusalem, Israel; 2 Department of General Surgery, Shaare Zedek Medical Center, Affiliated with the Hebrew University School of Medicine, Jerusalem, Israel; 3 Department of Obstetrics and Gynecology, Hadassah-Hebrew University Medical Center, Jerusalem, Israel; 4 Department of Obstetrics and Gynecology, Shaare Zedek Medical Center, 12 Bayit street, 91031 Jerusalem, Israel

**Keywords:** Appendicitis, Laparoscopy, Pregnancy, Negative-appendectomy

## Abstract

**Background:**

The impact on pregnancy of laparoscopy for acute appendicitis is well documented. However, with an accurate pre-operative diagnosis being more challenging in pregnant patients, the incidence of a negative appendectomy (NA) is higher in this cohort. The aim of this study was to evaluate the maternal and neonatal implications of a NA during pregnancy.

**Methods:**

A single center retrospective study between 2004 and 2019 was performed. Pregnant women who underwent laparoscopic appendectomy for suspected appendicitis were identified from which those who had a pathologically normal appendix were selected. The maternal and neonatal outcome of this group were compared with a matched control group of pregnant women who underwent diagnostic laparoscopy for a presumed ovarian torsion in whom no further surgical intervention was performed. Multivariate regression analysis was performed to explore factors that gestational size.

**Results:**

Of the 225 pregnant women who underwent laparoscopy appendectomy, a NA was performed in 33 (14.7%). These were compared with 50 pregnant women in the diagnostic laparoscopy group. The former was characterized by higher rate of nulliparity and later gestational age at the time of the surgery (17.8 ± 7.5 vs 11.3 ± 6.3, *p* < 0.001). Whilst the rate of maternal complications during pregnancy were similar between the groups, NA was associated with significantly lower neonatal birthweights (2733.9 ± 731.1 vs 3200.7 ± 458.5 g, *p* = 0.002) and a significantly higher risk of small for gestational age (SGA) infants (OR 5.6, 95% CI 1.02–30.9).

**Conclusions:**

Performing a NA during pregnancy is an indicator for perioperative counseling and antenatal follow up.

Acute appendicitis (AA) is the most common general surgical problem encountered during pregnancy [1]. The incidence of AA in pregnancy ranges between 1:1000 and 1:1400, similar to that of the non-pregnant population [2].

Laparoscopic appendectomy is the current recommended surgical approach for AA during pregnancy [3]. However, the safety of this approach during pregnancy especially in the first and third trimesters is still a subject of debate. Several large systematic reviews and meta-analyses have reported that laparoscopic appendectomy is associated with a higher incidence of fetal loss and reduced gestational age compared with open appendectomy [4–7]. It is thought that, in part, this is because the effects of the pneumoperitoneum and of carbon dioxide diffusion into the maternal bloodstream.

In order to avoid these complications, a pre-operative diagnosis is a vital part of the patient work-up in this specific cohort of patients. However, as a pre-operative diagnosis of AA in pregnant patients is more challenging, the incidence of a grossly normal appendix during diagnostic laparoscopy for a presumed AA is higher in pregnant compared to non-pregnant patients. Termed a negative appendectomy (NA), it has been found in up to 30% of cases [8, 9]. Nevertheless, as the grossly normal appendix can be pathologically inflamed, when no other abnormal pathology is found during laparoscopy for right iliac fossa pain, appendectomy is often performed [10].

Although the use of magnetic resonance imaging (MRI) has also been described in the academic literature with promising sensitivity alongside an acceptable safety profile [11], urgent MRI is not available in many hospitals, especially out-of-hours, thus NA is still common [12].

Whilst the impact of laparoscopy during pregnancy is well documented, the net impact of resecting a grossly normal appendix on maternal and neonatal outcomes has been less well explored. Therefore, the aim of this study was to explore the impact of a NA on maternal and neonatal outcomes.

## Material and methods

A retrospective study of prospectively collected data was performed in a the Shaare Zedek Medical Center. This tertiary hospital for antenatal care and general surgery is located in Jerusalem, Israel. Local ethics committee permission was sought and granted (0109-19-SZMC). Between January 2004 and January 2019, all consecutive women who underwent a diagnostic laparoscopy for a presumed diagnosis of AA were identified via a search of the hospital’s electronic medical records using the relevant codes for appendicitis or appendectomy. A second search within this cohort was performed to identify those patients who had a diagnosis of pregnancy at the time of surgery. In order to create a control group, an additional search was also performed to identify pregnant women who underwent diagnostic laparoscopy (DL group) during pregnancy for a presumed ovarian torsion.

The electronic notes of these patients were reviewed if they met the following inclusion criteria. For both groups, women had to have had a radiologically or biochemically confirmed pregnancy, must have been admitted urgently to undergo surgery and have been operated on laparoscopically without conversion to an open procedure. For the DL group, only those patients with a normal ovary without evidence of torsion were included if no further surgical intervention was performed. Pathology reports for patients in the NA group were also reviewed and only those patients with a pathologically normal and non-inflamed appendix were included in the study. Those patients with any other pathological findings were excluded from the study as were those with incomplete medical records.

All patients in our cohort had undergone thorough evaluation before surgery. A relevant clinical, gynecological and obstetric history including the progression of the current pregnancy, was taken. The patients' vital signs were recorded, and clinical examination findings noted, as were signs of uterine contractions or premature labor. Blood tests performed upon presentation to the ED were extrapolated. All women included in the study had an admission ultrasound study performed by a certificated medical technician or radiology specialist. Ultrasound findings suggestive of AA included: dilatation of the appendix ≥ 7 mm, appendix non-compressibility, edema of the appendiceal wall and local fat stranding. Women with a non-diagnostic ultrasound underwent a repeat delayed ultrasound assessment or a magnetic resonance imaging (MRI); the decision for a repeat imaging procedure was based on the clinical follow up and the magnitude of the clinical suspicion.

For the purpose of the study, gestational trimesters were classified as follows: The first trimester was from conception until week 14; the second trimester was from 15 until 23 weeks and 6 days gestation; and the third trimester was from 24 weeks gestation until delivery. Pre- and post-operative fetal viability was ascertained by ultrasound within 24 h of surgery. Small for gestational age (SGA) was defined as neonatal birthweights below the 10th centile for neonates of the same gestational age. Birth weight percentiles were based on Israeli live-born birth weight standards [13]. All surgical procedures were performed under general anesthesia using a pneumoperitoneum ranging between 12 and 15 mmHg.

From the files of the patients that were found to be eligible for inclusion in the study, demographic, obstetric, surgical and neonatal data were extracted. If women gave birth elsewhere, a telephone questionnaire was conducted by one of the research team using a standard script after patients gave informed consent for the questionnaire to be performed. Attempts were made to contact the patients six times with the contact information available in the hospital records. Patients were excluded if they were uncontactable, declined to partake in the study or who were unable to provide the information required.

### Statistical analyses

Statistical analysis was performed using SPSS version 21 (IBM SPSS Statistics for Windows, Version 23.0. Released 2012. Armonk, NY: IBM Corp). Descriptive statistics are described as mean or *N* with standard deviation or percentage in parenthesis unless stated otherwise. Univariate analysis was performed using either Chi-squared or Fisher’s exact test as appropriate. Continuous variables were analyzed using either an unpaired Student *T*-test or Mann–Whitney test as appropriate. A multivariate analysis was performed in a backward-stepwise manner on those variables found to be significant on univariate analysis using SGA as the dependent variable for those patients in the NA group. A *p* value of < 0.05 was considered statistically significant for the purposes of this study.

## Results

**Fig. 1:**
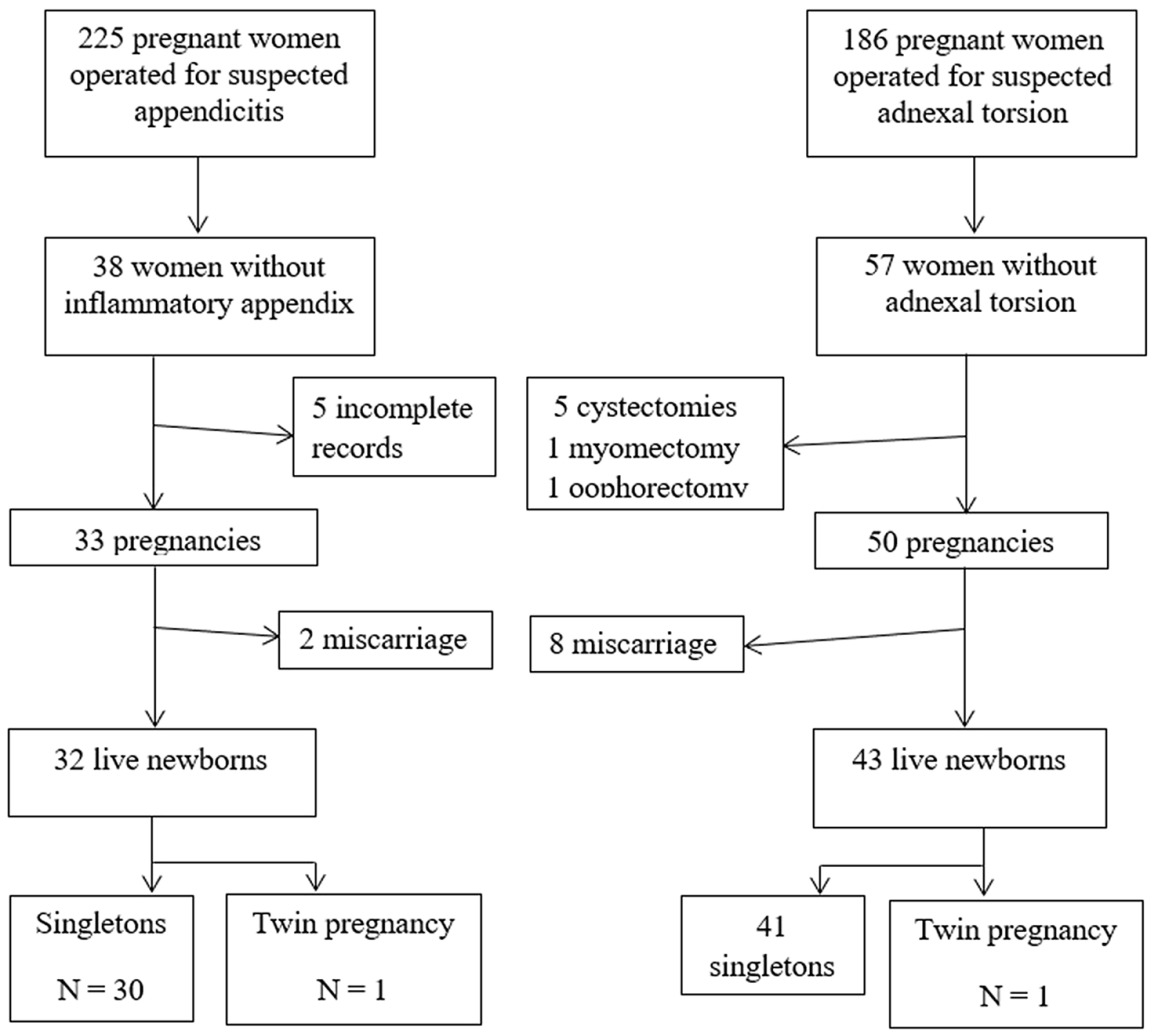
A flow diagram of patients involved in each of the two groups, intra-operative findings and the number of infants born into each cohort

A flow chart of the patient inclusion is displayed in Fig. 1. During the study period, 225 pregnant women underwent laparoscopic appendectomy for a suspected AA. Intra-operatively 189 patients (83.9%) were found to have macroscopic signs of appendicitis whilst 36 (16.1%) rest were grossly normal. Appendectomy was performed in all cases. On reviewing the pathology reports, 5 of the 189 cases (2.6%) that were found to be grossly inflamed intra-operatively had no signs of acute inflammation. Conversely, 3 of the 36 appendixes (8.3%) thought to be macroscopically normal were found to be pathologically inflamed. Therefore, from the initial cohort of 225 patients, 38 had a NA (16.9%) of whom 33 met the inclusion criteria and were ultimately included in the study.

During the study period, 186 pregnant patients were also identified who underwent laparoscopy for suspected adnexal torsion. In 57 patients (30.6%), there were no abnormal findings noted and no additional surgical procedure was performed. Once the inclusion criteria were applied, 50 patients were included in the DL group.

Demographic, clinicopathological and obstetric data of the study groups are described in Table 1. The NA group was characterized by significantly younger maternal age, lower parity and higher rate of nulliparity. Rates of prior cesarean deliveries, fertility treatments and a history of multiple gestations were comparable between the groups.

**Table 1:** A comparison of demographic and obstetric characteristics of the pregnant women included in the study

	Diagnostic laparoscopy (*N* = 50)	Negative appendectomy (*N* = 33)	*p* value
Maternal age (years)	28.7 ± 5.9	24.4 ± 4.3	< 0.001
Gravidity	3 [1–4.25]	2 [1–3.5]	0.07
Parity	1 [0–3]	0 [0–1.5]	0.04
Live children	1 [0–3]	0 [0–1]	0.03
Nulliparous	16 (32%)	18 (62.1%)	0.01
Previous cesarean delivery	11 (22%)	4 (13.8%)	0.38
Gestational week at surgery (weeks)	11.3 ± 6.3	17.8 ± 7.5	< 0.001
Gestational week at surgery ≤8 (weeks)	15 (30%)	3 (9.1%)	0.02
Trimester I	34 (68%)	11 (33.3%)	< 0.001
Trimester II	14 (28%)	20 (60.6%)	< 0.001
Trimester III	2 (4%)	2 (6.1%)	0.67
Multiple gestation	1 (2%)	1 (3%)	0.77
Fertility treatments	8 (16%)	2 (6.1%)	0.18

Data are presented as mean ± standard deviation, mean with interquartile range [IQR] or number (%)

Gestational age at surgery was also significantly different. Whilst in the NA group most women underwent surgery during the second trimester, in the DL group most women were operated on in the first trimester.

The comparison of maternal outcomes is described in Table 2. Whilst the overall incidence of miscarriage was lower in the NA group compared to the DL group, this difference failed to reach statistical significance (6.1% vs 16.0%, *p* = 0.18). No differences were noted with regards to other pregnancy related complications such as hypertensive disorders of pregnancy, gestational diabetes and placental abruption. Similarly, whilst the mean gestational age was lower and the rates of preterm delivery higher in the NA group, these differences approached but did not reach a level of statistical significance (36.5 ± 6.9 vs 38.6 ± 1.7, *p* = 0.07 and 12.9% vs 10%, *p* = 0.71 respectively). The mode of delivery was similar between the two groups.

**Table 2:** A comparison between the two groups of maternal outcomes

	Diagnostic laparoscopy (*N* = 50)	Negative appendectomy (*N* = 33)	*p* value
Miscarriage	8 (16%)	2 (6.1%)	0.18
Hospitalization with preterm labor	3 (6.3%)	4 (12.1%)	0.36
Hypertensive disorders of pregnancy	1 (2.5%)	0 (0%)	0.37
Gestational diabetes	1 (2.5%)	0 (0%)	0.37
Placental abruption	1 (2.5%)	1 (3.4%)	0.82
Gestational age at delivery (weeks)	38.6 ± 1.7	36.5 ± 6.9	0.07
Preterm rupture of membranes	0 (0%)	1 (3%)	0.27
Preterm delivery (< 37 weeks)	4 (10%)	4 (12.9%)	0.71
Mode of delivery			
Spontaneous vaginal delivery	29 (69%)	22 (71%)	0.86
Vacuum extraction	4 (9.5%)	1 (3.2%)	0.30
Cesarean delivery	9 (21.4%)	8 (25.8%)	0.67

Data are presented as mean ± standard deviation or number (%)

The neonatal outcomes are displayed in Table 3. There were 34 neonates born to the NA group and 51 to the DL group. Following NA, the mean neonatal birth weight was significantly lower (2733.9 ± 731.1 vs 3200.7 ± 458.5, *p* = 0.002) and the risk for neonatal SGA birth weight was higher (6 (20%) vs 2 (4.9%), *p* = 0.047). There were no cases of intra-uterine fetal death in either group.

**Table 3:** Neonatal outcomes

	Diagnostic laparoscopy (*N* = 50)	Negative appendectomy (*N* = 33)	*p* value
Apgar 1 min	8.6 ± 0.9	8.3 ± 2.1	0.43
Apgar 5 min	9.1 ± 0.5	8.7 ± 1.7	0.28
Neonatal birth weight (grams)	3200.7 ± 458.5	2733.9 ± 731.1	0.002
Small for gestational age (SGA, < 10th centile for population)	2 (4.9%)	6 (20%)	0.047

Data are presented as mean ± standard deviation or number (%)

An adjusted multivariate analysis substantiated the finding that NA during pregnancy was found to be independently associated with the risk of a SGA birthweight at any gestational age (OR 5.6, 95% CI 1.02–30.9).

## Discussion

In this retrospective study, we aimed to evaluate the maternal and neonatal outcomes amongst pregnant patients who underwent laparoscopic removal of a pathologically normal appendix. This was achieved by creating a comparative group of pregnant women who underwent a diagnostic laparoscopy for another common benign condition, torsion of the ovary. By selecting only those women who had normal intra-operative findings with the absence of any alternative intra-abdominal pathology, we were able to assess as closely as possible the impact of removal of a normal appendix. We found that throughout the study period, maternal outcomes were favorable. Nevertheless, laparoscopic appendectomy of non-inflamed appendix during pregnancy was associated with significantly higher rate neonates who were SGA irrespective of the gestational age at the time of birth.

Albeit the recently increased use of MRI for diagnosis of AA during pregnancy, negative appendicitis is still common in pregnancy [9, 11]. Additionally, urgent MRI is not available in many hospitals; thus in view of the difficulty of diagnosis, the unacceptability of conservative management and the serious impact of perforated appendicitis for the pregnancy, DL remains the preferred choice for cases where available resources prevent prompt advanced diagnostic imaging techniques or in case investigations are inconclusive [3, 12].

In our cohort NA rate was 16.9% (38/225) which is lower as compared to the rates reported by others (22–30%). While previous studies reported a higher incidence of NA in pregnant as compared to non-pregnant patients, others have shown a similar rates in both groups (23 and 22%, *p* = 0.9), [14]. An equivalent rate of NA may be explained by the greater utilization of any preoperative imaging, the inter-user dependency of US and increased use of MRI.

It has previously been shown that when evaluating the neonatal outcomes following surgery performed for a presumed AA, resection of a grossly normal appendix is associated with increased rates of preterm labor and fetal loss [15, 16]. However, these studies compared NA to removal of an inflamed appendix and thus, may reflect the effect of intra-abdominal inflammation on neonatal outcomes. The present study, to the best our knowledge, is the first to explore these outcomes in the absence of intra-abdominal inflammation.

In the cohort presented here, 8.3% of appendixes that were found to be macroscopically normal intraoperatively were in fact found to be inflamed on pathological examination. That the grossly normal appendix may ultimately be inflamed underpins the practice of some surgeons to remove a normal appearing appendix if no other intra-abdominal findings are noted during surgery [10, 17]. Antibiotic therapy has been proposed as an alternative to surgery for the treatment of AA. In a large recent multicenter randomized trial, antibiotics were found to be noninferior to appendectomy [18]. In the setting of pregnant women in whom the appendix is macroscopically grossly normal during surgery, the option of antibiotic therapy without removing the appendix may be considered. Although a high relapse rate of 29% has been found, it could allow enough time to allow the pregnancy to continue to term. In parallel, one has to consider the significant risks of maternal and fetal morbidity and mortality, should surgery be performed later in pregnancy. Further studies exploring this potential treatment modality among the pregnant population with AA are warranted.

Albeit with initial hesitation, laparoscopic surgery in pregnancy has now become an accepted standard of care. Although concerns existed regarding the maternal and neonatal outcome as well as expected technical difficulties, later publications showed laparoscopy to be safe for both mother and fetus [3]. Nevertheless, meta-analyses have shown that compared to laparotomy, laparoscopy may be associated with increased rates of fetal loss and early gestational age at the time of birth [5, 6]. Our current study findings are in line with the more recent meta-analysis, showing no higher rate of miscarriage and preterm labor associated with laparoscopic appendectomy [19].

Interestingly, whilst the rate of preterm delivery did not differ between the groups, in the NA group neonatal birthweights were significantly lower and rates of SGA significantly higher. To the best of our knowledge, this relationship has not been previously delineated in the academic literature. A possible explanation for this trend could be that the local inflammation that occurs following the resection of even a normal appendix may cause a degree of intra-uterine growth retardation [20]. These findings are of clinical importance. Firstly, it provides an evidence based approach to the decision to remove the grossly normal appendix. Secondly, it should be considered in the consenting process when performing laparoscopic appendectomy in pregnant patients. Finally, despite the fact that some grossly normal appendixes will harbor pathological inflammation, for those patients at higher risk of SGA or low birthweight infants consideration should be given to avoiding resection of the grossly normal appendix. The cause of this this relationship warrants further investigation in future studies.

There are some limitations to this study. Firstly, the retrospective nature of this study may result in bias towards data collection and reporting. In addition, relevant variables may have been excluded from the analysis such as non-intra-abdominal causes of abdominal pain or a maternal history of SGA and preterm delivery. Secondly, all subjects underwent laparoscopic surgery and therefore we cannot comment on the impact of open procedures. Finally, the results found here are based on a relatively small sample size cohort and as such the multivariate analysis was limited in the number of variables controlling for the risk for SGA and it's is possible that in a larger sample size cohort, while controlling for additional variables, the association between NA and SGA will be annulled. Additionally, the study may be underpowered to draw conclusions for infrequent outcomes such as intra-uterine fetal death. On the other hand, the main strengths of our study include its unique cohort of pregnant women underwent DL and the meticulous data collection.

In conclusion we have shown an overall favorable perinatal outcome following a NA. However, NA was independently associated with higher rates of SGA infants and low birth weights. Due to the small number of patients in the study group, further studies are needed to understand the relationship between NA and neonatal birth weight. Nevertheless, considering the significant association shown, we suggest that this information may be in the pre-operative counseling process in this subset of women and the immediate postoperative antenatal follow-up of women with NA during pregnancy.
